# Efficacy of salicylic acid chemical peeling in treating a case of severe *Demodex folliculorum-induced* rosacea

**DOI:** 10.1016/j.jdcr.2026.04.049

**Published:** 2026-04-30

**Authors:** Yujiang Li, Xu Liu, Gang Wang, Xian Jiang

**Affiliations:** a Department of Dermatology and Venerology, Sichuan University West China Hospital, Chengdu, Sichuan, China; b Department of Dermatology, Sanmenxia Central Hospital of Henan University of Science and Technology, Henan University, Sanmenxia, Henan, China; c Frontier Science Center for Disease Molecular Networks, Sichuan University West China Hospital, Chengdu, Sichuan, China

**Keywords:** Demodex, reflectance confocal microscopy, rosacea, salicylic acid chemical peeling

## Introduction

Rosacea is a common chronic inflammatory skin disorder, characterized by central facial erythema, papules, pustules, telangiectasia, etc.^1^ Its pathogenesis remains incompletely understood, involving genetic, immunologic, neurogenic, vascular, and environmental factors.^2^ Recently, increasing evidence has confirmed the important role of Demodex infestation in rosacea development. *Demodex folliculorum* mites reside in pilosebaceous units, inducing perifollicular inflammation that exacerbates rosacea.^3^^,^^4^

Conventional therapies are sometimes insufficient for Demodex-associated rosacea, particularly in refractory cases. In recent years, salicylic acid peeling has been increasingly used in dermatologic practice due to its keratolytic, antibacterial and anti-inflammatory properties. However, few reports exist on its efficacy specifically for rosacea with severe demodicosis. We present a recalcitrant case of rosacea complicated by heavy Demodex infestation, successfully managed with salicylic acid peeling. Dynamic reflectance confocal microscopy (RCM) observation provided in vivo visualization of mite clearance. This may offer reference for diagnosing and treating similar patients.

## Case report

### History and examination

**Fig 1:**
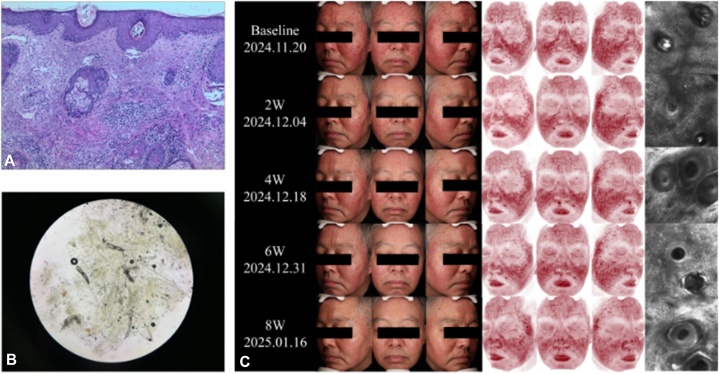
Patient's clinical presentation and ancillary tests (**A,** Histopathology showing follicular *Demodex folliculorum* mites; **B,** Microscopy revealing numerous Demodex bodies; **C,** Facial clinical appearance and dynamic RCM images throughout treatment course). *RCM*, Reflectance confocal microscopy.

A 59-year-old male sought treatment for a 2-year history of pruritic facial erythema and papules. The rash first appeared without obvious triggers, accompanied by intense itching. He was previously diagnosed with rosacea and treated with antibiotics, corticosteroids, and other therapies at other hospitals, with poor response and recurrence. He denied a history of drug or cosmetic allergies, urticaria, or allergic rhinitis. Examination revealed diffuse facial erythema and papules, some coalescing into plaques, with yellowish-white scales and sebaceous plugs. Scattered erythematous papules and pustules resembled grains of rice. The involved skin felt rough on palpation (Fig 1, *C*).

### Laboratory tests

CBC: WBC 9.7 × 10ˆ9/L, neutrophils 83.7%, lymphocytes 8.1%. Liver and kidney function, lipids, and fasting glucose were normal. ESR was elevated at 35 mm/h. ANA was positive at a titer of 1:640. Lesional skin biopsy showed numerous *D folliculorum* mites within follicles and lymphocytic infiltration around superficial blood vessels and pilosebaceous units in the dermis (Fig 1, *A*). Microscopy of follicles revealed abundant Demodex bodies (Fig 1, *B*).

### Diagnosis and differential diagnosis

Based on clinical presentation, histopathology and mite microscopy, the diagnosis was as follows:

(1) Rosacea, papulopustular, stage II. The patient had persistent facial erythema, papules and pustules with sebum excretion, consistent with rosacea diagnosis considering the disease course and histologic findings.^5^ Clinical findings indicated moderate-to-severe stage II papulopustular.^5^
(2) Severe demodicosis. Biopsy revealed abundant follicular *D folliculorum*, confirmed by microscopy. Combined with clinical features, severe demodicosis was diagnosed.
(3) Differential diagnoses included:
  ① Rhinophyma, usually involving the nose with predominant erythema and papules, atypical of this case's diffuse distribution with pustules and pruritus;
  ② Seborrheic dermatitis, presenting as facial erythema and scaling without papulopustules, and no mites on microscopy;
  ③ Discoid lupus erythematosus, but histology showed no interface dermatitis, ANA titer was not accompanied by typical clinical features, and lesions lacked classic discoid plaques.

### Treatment and course

Considering the severe presentation, poor response to standard therapies, and confirmation of heavy follicular demodicosis by biopsy and microscopy, 30% salicylic acid chemical peeling was administered. The face was cleansed, Vaseline was applied around the eyes and mouth, and 30% salicylic acid was applied to the lesions until a burning sensation occurred, then immediately removed. Starting at 15 minutes, application time was gradually increased to 30 minutes. Treatments were performed every 2 weeks for a total of 4 sessions. Moisturizers were used between sessions.

After treatment, facial erythema and papules largely resolved and pruritus was markedly relieved (Fig 1, *C*). RCM follow-up showed that at baseline, follicular dilation, spongiosis, papillary dermal vessel dilation and inflammation were present, with approximately 10 mites visible per field of view. After treatment, inflammation subsided, follicular structure normalized, and no motile mites were observed. The patient was very satisfied with the outcome. Subsequent IPL treatment further improved residual erythema and hyperpigmentation. No significant recurrence—defined as the reappearance of persistent erythema, papules, or pustules requiring medical intervention—was observed during the 2-month follow-up after cessation of salicylic acid peeling.

## Discussion

Demodex mites are tiny arthropods that parasitize human pilosebaceous units. They are ubiquitous in the general population and usually asymptomatic. However, in susceptible individuals, such as the elderly or those with altered immune status, Demodex overgrowth can trigger inflammation and contribute to various dermatoses.^1^^,^^4^ Increasing evidence has confirmed the close association between demodicosis and rosacea development and progression. A meta-analysis by Chang et al showed significantly higher Demodex prevalence and density in rosacea patients compared to healthy controls.^2^ Studies conducted in China have reported Demodex positivity rates of 60% to 80% in patients with rosacea.^6^^,^^7^

Notably, this patient presented with elevated ESR and ANA levels, suggesting possible underlying immune dysregulation. Although no specific autoimmune disease was diagnosed and no targeted intervention was required, such immune alterations may contribute to Demodex proliferation and increased susceptibility to inflammation. This highlights the importance of considering host immune status in patients with refractory rosacea.

Standard treatments for demodicosis, such as oral ivermectin and topical antimite medications, are generally effective; however, recurrence may occur in some patients.^1^^,^^8^

In recent years, salicylic acid peeling has emerged as a potential therapeutic approach in dermatology. It has been widely used in acne treatment due to its keratolytic and anti-inflammatory effects,^9^ which may also be beneficial in rosacea by reducing follicular plugging, controlling inflammation, and improving skin barrier function.

This patient received 30% salicylic acid peeling every 2 weeks for 4 sessions, with marked clinical improvement. RCM confirmed near-complete elimination of Demodex mites from follicles. This suggests that salicylic acid peeling may be a safe and effective option for refractory rosacea with severe demodicosis. Potential mechanisms include removal of follicular plugging, enhancement of topical penetration, and direct mite eradication. Previous international studies have suggested that salicylic acid may exhibit miticidal effects by disrupting the mite exoskeleton and inhibiting respiration.^10^^,^^11^

RCM is an emerging noninvasive imaging technique that allows real-time visualization of skin microstructure.^12^ It has gained increasing attention for its role in diagnosing and monitoring demodicosis. Sattler et al demonstrated that RCM can quantify Demodex density with high concordance to traditional microscopy.^13^ González et al further confirmed its value in evaluating Demodex-related dermatoses.^12^ In this case, dynamic RCM observation objectively demonstrated mite clearance following treatment. Importantly, RCM enabled real-time visualization of mite clearance, providing objective in vivo evidence supporting the therapeutic efficacy of salicylic acid peeling.

In summary, this case suggests that Demodex-targeted therapies should be considered in refractory rosacea. Salicylic acid chemical peeling may represent an effective and safe alternative therapeutic option. Noninvasive imaging modalities such as RCM can assist in diagnosis and allow real-time monitoring of treatment response. Larger prospective studies are warranted to further validate these findings.

## Conclusion

This report describes a patient with treatment-resistant rosacea due to severe demodicosis. After salicylic acid chemical peeling, skin lesions and symptoms markedly improved, with RCM confirming near-complete mite clearance. This indicates that the treatment is safe and effective for Demodex-associated rosacea. For patients with refractory or recurrent rosacea, particularly those with potential immune dysregulation, Demodex infestation should be considered. Special examinations such as RCM can facilitate diagnosis and guide personalized management. Larger prospective studies are still needed to further validate its clinical utility.

## Ethical considerations

This case report was conducted in accordance with the principles outlined in the Declaration of Helsinki. The patient provided written informed consent for the publication of this report and accompanying images.

## Conflicts of interest

None disclosed.
